# Medicinal plant-derived bioactive compounds modulate gut microbiota–host signaling in cancer-related fatigue: a phenotype-to-target framework

**DOI:** 10.3389/fmicb.2026.1988968

**Published:** 2026-10-23

**Authors:** Yu Jiao, Qin Feng, Yifan Ren, Jinni Sun, Ruyu Wu, Qingchuan Li, Jingzhe Gao, Zhengcan Zhou, Qi Qiu, Zhigang Wang, Kunshuang Shen, Ning Zhang, Liang Zheng, Hui Sun

**Affiliations:** 1 State Key Laboratory of Integration and Innovation of Classic Formula and Modern Chinese Medicine, National TCM Key Laboratory of Serum Pharmacochemistry, Metabolomics Laboratory, Department of Pharmaceutical Analysis, National Chinmedomics Research Center, College of Pharmacy, Heilongjiang University of Chinese Medicine, Harbin, China; 2 Shandong Key Laboratory of Gelatine Medicines Research and Development, Dong’e Ejiao Co., Ltd., Liaocheng, China

**Keywords:** cancer-related fatigue, gut microbiota, gut–brain communication, immune–metabolic signaling, medicinal plant-derived bioactive compounds, plant–microbiota–host axis, short-chain fatty acids, tryptophan metabolism

## Abstract

Cancer-related fatigue (CRF) is a frequent and disabling symptom during cancer and anticancer therapy, and pathway-directed pharmacological treatment remains limited. This review examines CRF through the plant–microbiota–host axis, in which medicinal plant-derived bioactive compounds reshape gut microbial community structure and function and are in turn biotransformed by gut microbes into metabolites that host sensors detect. Tumor- and treatment-associated injury of the intestinal mucosa alters epithelial renewal, barrier selectivity, and microbial metabolism, reshaping host exposure to microbial products and metabolites. These changes generate molecular mediators, including short-chain fatty acids, tryptophan-derived metabolites, bile acids, and microbe-associated molecular patterns, which are detected by epithelial, immune, enteroendocrine, and neural sensors. We organize the evidence into mediator–sensor modules and discuss how vagal, humoral, and immune–metabolic routes may converge on brainstem and monoaminergic networks involved in activity, effort, and interoceptive malaise. Because direct causal evidence in CRF remains incomplete, we separate established biology from candidate mechanisms and define pharmacodynamic readouts that can test target engagement. Medicinal plant-derived compounds enter the framework upstream: their microbial conversion products engage the same mediator–sensor modules. We argue that future CRF studies should match the fatigue domain, biological subgroup, molecular target, and biomarker before efficacy is interpreted mechanistically; for plant-derived interventions, this matching must also include the administered compound and its microbially generated metabolites.

## 1 Introduction

Cancer-related fatigue (CRF) differs clinically from ordinary tiredness. It can persist despite rest and can involve perceived effort, spontaneous activity, endurance, motivation, and cognitive–mood engagement. Its burden during treatment and survivorship is well described, but therapy has advanced more slowly than epidemiology (Bower, 2014; Ryan et al., 2007; Saligan et al., 2015; Bower, 2019). Inflammation, hypothalamic–pituitary–adrenal disruption, mitochondrial injury, sleep disturbance, anemia, cachexia, and mood symptoms all contribute in specific settings (Dantzer et al., 2008; Karshikoff et al., 2017; Kang et al., 2023). These processes are often listed in parallel rather than connected into a testable mechanism. A molecular account should ask a narrower set of questions: what signal is sensed, which cell detects it, which route carries it, and which fatigue domain is affected. Current guidance gives appropriate priority to exercise, psychological, and integrative approaches; drug options remain limited and context-dependent (Bower et al., 2024; Berger et al., 2015; Mustian et al., 2017). Psychostimulants and corticosteroids may help selected patients, but their effects are not sufficiently durable, specific, or safe to define a general CRF pathway (Stone et al., 2024; Yennurajalingam et al., 2013). The association between the systemic immunity-inflammation index and CRF therefore supports inflammation as a useful stratification variable, but not as a complete explanation of fatigue (Sun et al., 2025).

The gut is a plausible entry point because it is not merely a biomarker source; it is a sensing surface. The intestinal mucosa contains rapidly renewing epithelial cells, mucus and tight-junction systems, resident immune cells, enterochromaffin and other enteroendocrine cells, microbial communities capable of fermentation and xenobiotic transformation, and vagal and spinal sensory terminals near the lamina propria. Cancer therapy and cancer-related stress physiology can disturb this interface. When barrier selectivity is weakened, microbial products and metabolites are no longer confined to the lumen; they become signals available to host sensors. Here, gut-derived immune–metabolic signaling refers to the sequence of mucosal injury, mediator generation, host detection, and gut-to-brain communication. The relevant question is not whether one taxon is higher or lower in a fatigued cohort, but whether gut-derived molecules engage a measurable sensor system that can be modified and linked to a defined fatigue domain.

The evidentiary hierarchy is deliberately conservative. Direct CRF studies are weighted most heavily, followed by cancer or cancer-treatment models with fatigue-relevant behavior, non-cancer inflammatory models, and anatomical or receptor-expression studies that support plausibility without establishing causality. Gut dysbiosis in fatigued patients, mucositis biology, short-chain fatty acid (SCFA) signaling, vagal immune sensing, and dorsal raphe nucleus (DRN) serotonin physiology each have supporting literature, but they do not yet form a demonstrated causal chain in CRF. Existing CRF reviews have usually emphasized one compartment at a time: peripheral inflammation, hypothalamic–pituitary–adrenal (HPA) axis dysregulation, skeletal muscle mitochondrial dysfunction, psychosocial factors, or exercise interventions. Microbiome-focused cancer reviews have often stopped at taxonomic associations. This review treats the gut as a sensory interface in which epithelial injury, microbial metabolism, and host detection can be connected through defined molecular pathways. It separates gut-derived signals into mediator–sensor modules and uses a route-dominance model rather than assigning all effects to a single gut–brain pathway. Each proposed target is paired with the type of result that would weaken the mechanistic claim. This keeps the argument anchored in testable biology rather than descriptive association.

This review develops a molecular framework for how medicinal plant-derived bioactive compounds influence the plant–microbiota–host axis in CRF. These compounds may act through three non-exclusive routes: reshaping gut microbial community structure and function, undergoing microbiota-dependent biotransformation, and acting directly on host targets; this review concentrates on the two microbiota-dependent routes. The central question is not whether a microbial taxon is increased or decreased in fatigued patients, but whether plant-derived compounds, through microbial remodeling or biotransformation, generate molecules that engage defined host sensors and communication routes linked to a specific fatigue domain. We therefore organize the literature around mediator–sensor modules—SCFAs, tryptophan-derived metabolites, bile acids, microbe-associated molecular patterns (MAMPs), and gut sensory-cell signals—each of which can be engaged by plant constituents through their microbial metabolism. We then evaluate how these signals may reach the nervous system through vagal, humoral, and immune–metabolic routes and where they may converge in brainstem and monoaminergic networks. Throughout the review, established molecular biology is separated from candidate CRF mechanisms, and each proposed pathway is paired with the type of pharmacodynamic evidence needed to support or weaken a causal interpretation.

## 2 Review scope and evidence appraisal

This is a narrative review focused on molecular and translational mechanisms rather than a systematic review or meta-analysis. Evidence was considered in four levels: direct CRF studies with fatigue-specific endpoints; cancer or cancer-treatment models with fatigue-relevant behavior; mechanistic studies of mucosal injury, microbial metabolites, and gut-to-brain signaling in non-cancer inflammatory models; and anatomical or receptor-expression studies that support biological plausibility. Targeted searches of PubMed and Web of Science (updated through September 2026) were used to identify studies on cancer-related fatigue, gut microbiota, intestinal barrier injury, microbial metabolites, medicinal plant-derived bioactive compounds and their microbial biotransformation, vagal and humoral signaling, nucleus tractus solitarius (NTS) and DRN biology, serotonin, kynurenine metabolism, and gut–brain communication. Searches were organized around four concept blocks: cancer-related fatigue, gut microbiota and intestinal barrier, medicinal plant-derived compounds and their microbial metabolism, and gut–brain signaling. PRISMA procedures were not applied. Positive and negative findings are therefore interpreted through an evidence hierarchy rather than as pooled estimates. Mechanistic claims are treated as strongest when a defined molecular signal, host sensor, communication route, fatigue domain, and pharmacodynamic readout are linked in the same experimental or clinical framework. Figure 1 summarizes this working model and shows how tumor- and treatment-associated stressors may be connected to mucosal sensing, parallel gut-to-brain routes, central integration, fatigue domains, and target-engagement readouts.

**Figure 1:**
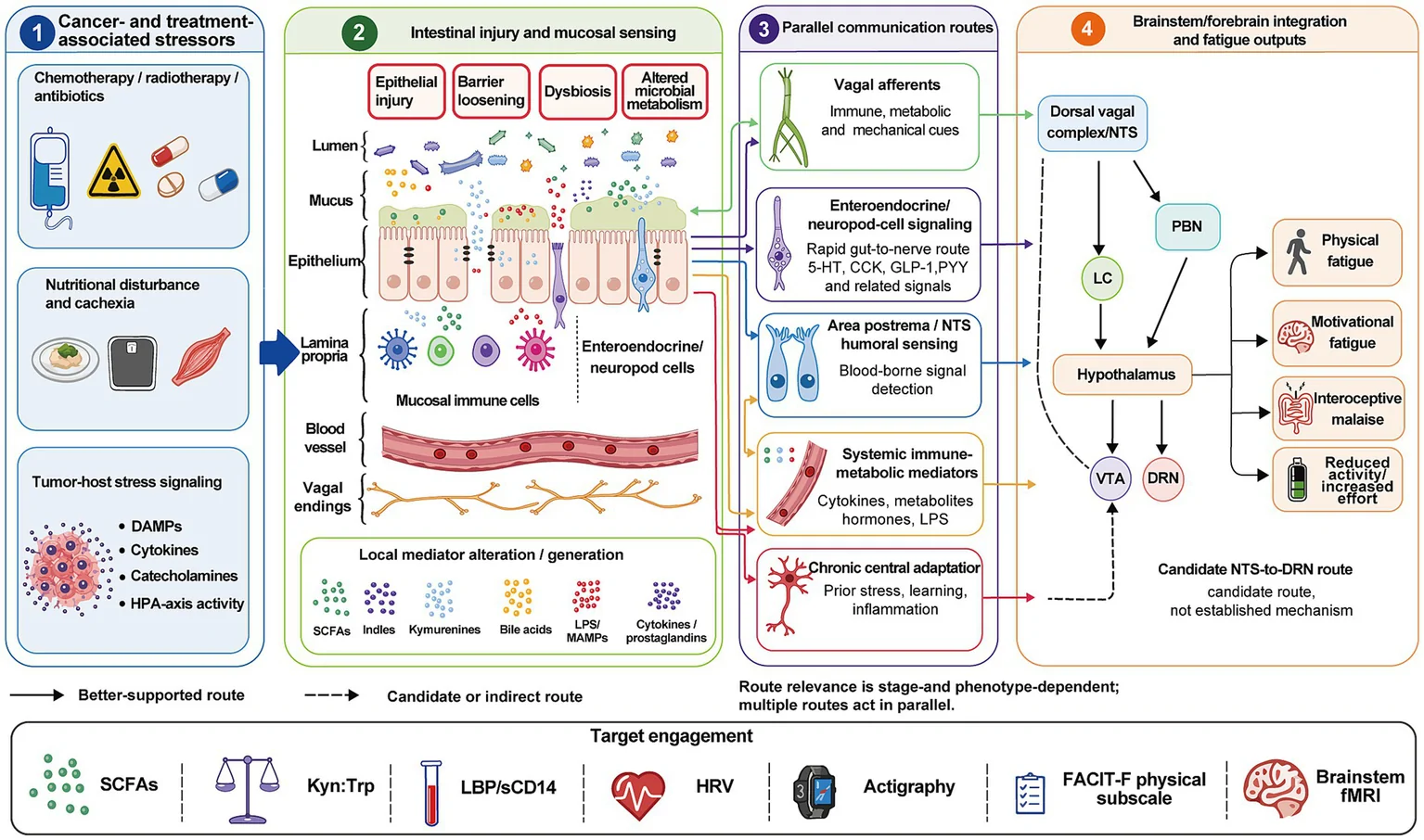
Molecular model of gut-derived immune–metabolic signaling in CRF. Cancer- and treatment-associated stressors can injure the intestinal epithelial interface, alter microbial metabolism, and increase host exposure to microbial products and metabolites. These signals are detected by mucosal immune, endocrine, and neural sensors and transmitted through parallel vagal, enteroendocrine/neuropod-cell, humoral, immune–metabolic, and central-adaptation routes. Brainstem and forebrain nodes then integrate these inputs to regulate activity, effort, and interoceptive malaise. Solid arrows indicate better-supported routes; dashed arrows indicate candidate or indirect routes requiring validation. Medicinal plant-derived bioactive compounds enter this model upstream, through microbiota remodeling and microbial biotransformation into the metabolites sensed at the mucosal interface.

Searches were conducted in PubMed and Web of Science in September 2026. Search terms were organized into four conceptual blocks covering cancer-related fatigue; gut microbiota and intestinal barrier injury, including mucositis; medicinal plant compounds and their microbial metabolism; and gut–brain signaling. When searched separately in PubMed, these four term blocks retrieved 2,741, 155,322, 11,855, and 25,559 records, respectively. However, overlap with the cancer-related fatigue literature was limited: 26 records addressed cancer-related fatigue together with gut microbiota or barrier injury, 10 addressed cancer-related fatigue in relation to gut–brain signaling, and only one addressed cancer-related fatigue together with medicinal plant compounds or their microbial metabolism. Much of the mechanistic evidence discussed in this review therefore derives from adjacent fields, and CRF-specific evidence is distinguished from indirect mechanistic evidence throughout. Titles and abstracts were independently screened by Y. J. and Q. F., with disagreements resolved by H. S. Full texts were subsequently assessed for relevance and inclusion in the narrative synthesis.

The review was developed with reference to the Scale for the Assessment of Narrative Review Articles (SANRA), particularly its emphasis on the justification of literature selection and the balanced presentation of conflicting evidence.

## 3 CRF phenotypes: Which fatigue dimension is biologically addressed?

A mechanistic account of CRF must specify the fatigue domain under study. Patient-reported fatigue includes physical, motivational, cognitive, affective, and sleep-related components. These domains often correlate clinically, but they should not be treated as interchangeable. Physical fatigue maps most directly onto reduced activity, reduced endurance, and increased effort. Motivational fatigue concerns the willingness to initiate or sustain goal-directed behavior. Cognitive fatigue involves attention, working memory, and executive function, whereas sleep-related fatigue overlaps with insomnia, circadian disruption, pain, corticosteroid exposure, and sedating medication. Gut-derived immune–metabolic signaling is most plausible for physical and motivational fatigue because both are closely tied to interoceptive malaise, inflammatory sensing, autonomic state, and activity suppression (Phan et al., 2024; Vichaya et al., 2023). Animal models make this distinction sharper. Voluntary wheel running, home-cage activity, open-field distance, and treadmill endurance measure activity or capacity, but they are not direct homologues of the human symptom. Effort-based choice, progressive-ratio responding, burrowing, and nesting can sample motivational components if locomotion, reward value, anxiety-like behavior, food intake, and sickness are controlled. In recent cancer-model work, tumor burden and therapy reduced activity without a detectable motivational deficit, and associative learning helped maintain fatigue-like behavior after the initiating inflammatory signal had subsided (Phan et al., 2024; Vichaya et al., 2023). These observations argue against composite fatigue-like scores that merge activity, anxiety, sleep, and appetite.

The same principle applies in clinical trials. A probiotic, fermentable fiber, barrier-protective agent, or formula may improve physical fatigue when mucosal inflammation and microbial metabolite loss are active; it is less likely to improve attention when sleep fragmentation or chemotherapy-related cognitive impairment is the dominant driver. A vagal intervention is more rational in patients with low heart-rate variability (HRV), gut injury, and inflammatory symptoms than in anemia-dominant fatigue without autonomic dysregulation. Several common fatigue readouts are also confounded: reduced open-field distance may reflect anxiety, neuropathic pain, cachexia, motor weakness, or sedation; reduced wheel running may reflect learned aversion, altered reward value, or musculoskeletal toxicity rather than central fatigue; and in patients, fatigue severity may track sleep disturbance, depression, anemia, endocrine therapy, or opioid exposure. These variables are not merely nuisances for statistical adjustment; they are competing biological explanations. A credible molecular model should predict which covariates modify response and which require exclusion or stratification.

A practical outcome structure has three layers: a domain-specific fatigue scale, an objective activity or performance measure, and a pathway-specific pharmacodynamic readout. A gut-barrier intervention cannot be interpreted from the Functional Assessment of Chronic Illness Therapy–Fatigue (FACIT-F) total score alone; it requires physical subscales, actigraphy, gastrointestinal toxicity, permeability or translocation markers, and inflammatory-metabolic biomarkers. Vagal interventions need HRV or related autonomic indices, whereas kynurenine-axis studies need targeted metabolite panels. Such a structure does not guarantee efficacy, but it reduces mechanistic overinterpretation. Table 1 maps major fatigue domains to clinical endpoints, preclinical approximations, plausible gut-derived mechanisms, trial-design implications, and representative evidence. The aim is not to assign a single mechanism to each symptom, but to identify the domains in which gut-derived immune–metabolic signaling is biologically plausible. This phenotype-first structure is essential for molecular interpretation because the same mediator may affect activity, effort, sleep, or appetite through different host sensors and neural routes.

**Table 1:** Phenotype-to-mechanism mapping for gut-derived immune–metabolic signaling in CRF.

Fatigue dimension	Clinical presentation and preferred endpoints	Preclinical approximation	Most plausible gut-derived contribution	Trial implication	Representative evidence
Physical fatigue	FACIT-F physical items, BFI severity/interference, actigraphy, reduced daily activity and endurance.	Home-cage activity, voluntary wheel running, open-field distance with sickness and anxiety controls, treadmill/endurance tasks.	Barrier injury, SCFA loss, mucosal cytokine/prostaglandin signaling, lipopolysaccharide (LPS) exposure, altered vagal and area postrema/NTS input.	Best primary domain for barrier-, SCFA-, microbiome- or vagus-targeted interventions; require activity and GI/toxicity covariates.	Bower (2014), Bower et al. (2024), Phan et al. (2024), Vichaya et al. (2023), Grant et al. (2021), and Lai et al. (2026)
Motivational fatigue	Effort items, apathy-like symptoms, reduced task initiation, reduced willingness to sustain demanding behavior.	Progressive-ratio responding, effort-based choice, nesting/burrowing with locomotor and reward-value controls.	Inflammation-induced changes in interoceptive gain and monoamine circuits; NTS-PBN-LC-hypothalamic and VTA/DRN interfaces may contribute.	Use effort-specific endpoints. Low locomotion alone cannot be interpreted as motivational fatigue.	Dantzer et al. (2008), Karshikoff et al. (2017), Phan et al. (2024), and Vichaya et al. (2023)
Cognitive fatigue	PROMIS cognitive fatigue, FACT-Cog, attention and executive complaints.	Y-maze, novel object recognition, set-shifting, 5-choice serial reaction time.	Possible influence from kynurenine metabolites, systemic inflammation and sleep disturbance; vagal specificity is weaker.	Treat as secondary unless cognitive testing and central biomarkers are built into the design.	Bower (2014), Saligan et al. (2015), Kang et al. (2023), and Sun et al. (2025)
Sleep/circadian fatigue	PSQI, insomnia scales, actigraphy, sleep diary, daytime sleepiness.	EEG/EMG sleep staging, circadian wheel rhythm, body-temperature rhythm.	Inflammation and gut signals may affect arousal systems, but pain, steroids, circadian disruption and medications often dominate.	Analyze separately; do not attribute sleep-driven fatigue to gut–brain signaling without sleep measures.	Bower (2014), Kang et al. (2023), Bower et al. (2024), and Sun et al. (2025)
Anemia/cachexia-related fatigue	Hemoglobin decline, weight loss, muscle wasting, anorexia, inflammatory cachexia features.	Body composition, food intake, tumor burden, hemoglobin, muscle force.	Gut can contribute through nutrition and inflammation, but hematopoietic and muscle-catabolic mechanisms may dominate.	Stratify or exclude when testing gut-sensory pharmacology; relevant to hematopoietic or multi-component supportive interventions only when hematological endpoints are measured.	Bower (2014), Yennurajalingam et al. (2013), and Okinaka et al. (2024)

In this table support phenotype definition, endpoint selection, or major competing mechanisms. They are representative rather than exhaustive and do not provide direct evidence for every gut-derived mechanism listed in the table. BFI, brief fatigue inventory; PBN, parabrachial nucleus; LC, locus coeruleus; VTA, ventral tegmental area; PROMIS, patient-reported outcomes measurement information system; FACT-cog, functional assessment of Cancer therapy–cognitive function; PSQI, Pittsburgh sleep quality index; EEG, electroencephalography; EMG, electromyography; GI, gastrointestinal.

## 4 The injured intestine as a molecular sensory interface

Clinical CRF microbiome studies have been important because they moved the field beyond isolated cytokine measurements. Pelvic radiotherapy cohorts, advanced cancer cohorts, and lung cancer chemotherapy cohorts have reported associations between fatigue severity and microbial diversity, butyrate-producing taxa, Enterobacteriaceae enrichment, or microbial functional potential (Wang et al., 2015; Hajjar et al., 2021; Xiao et al., 2021; Wei et al., 2023; Slack et al., 2024). Systematic reviews reach a similar conclusion: associations are present, but they are heterogeneous, often cross-sectional, and confounded by tumor type, treatment, antibiotics, diet, opioids, proton-pump inhibitors, performance status, and gastrointestinal toxicity (Song and Bai, 2021). Recent breast-cancer and pediatric oncology data strengthen the association while remaining largely non-causal: gut microbiota statistically mediated the association between dietary quality and CRF in one breast-cancer cohort, and multi-omics during pediatric chemotherapy linked microbiome-metabolome pathways with a symptom cluster that included fatigue (He et al., 2024; Bai et al., 2024). A pediatric oncology review similarly supports integrating microbiome and metabolic pathways when interpreting treatment-related psychoneurological symptoms (Webster et al., 2024). Small cohorts require caution. Depletion of Faecalibacterium, Roseburia, or Lachnospiraceae may suggest reduced butyrate-producing capacity, but function cannot be inferred securely without metagenomic or metabolomic data. Enrichment of Enterobacteriaceae may indicate inflammatory potential, but host exposure still depends on barrier integrity, mucus thickness, antimicrobial peptides, and immune containment. Taxonomy is most useful as a hypothesis generator, and microbiome studies should report sampling, sequencing, metadata, and analysis choices with sufficient transparency for interpretation (Mirzayi et al., 2021).

Timing and function matter as much as composition. A sample taken before chemotherapy may capture risk; a sample taken during mucositis may capture active signal generation; and a sample taken after treatment may capture recovery or persistence. Pooling these states can obscure the difference between risk, active injury, and repair. In acute treatment, mucosal injury may amplify microbial sensing even when dysbiosis is modest. In survivorship, fatigue may persist after mucosal healing, in which case gut findings may be secondary, compensatory, or unrelated. In non-cancer chronic fatigue research, the most consistent signal is not a single organism but reduced butyrate-producing capacity and disturbed microbial metabolic networks. In cancer, a similar functional loss becomes mechanistically relevant only when it coincides with epithelial injury, inflammatory sensing, and a measurable fatigue phenotype. Stronger studies will therefore pair taxonomy with gene pathways, metabolites, barrier indices, and patient-level covariates such as antibiotics and diet (Guo et al., 2023; Xiong et al., 2023).

Mucositis biology provides the host-side context. Cytotoxic therapy can induce reactive oxygen species, DNA damage, NF-κB activation, epithelial apoptosis, crypt dysfunction, tight-junction disruption, ulceration, and secondary microbial amplification (Sonis, 2004; van Vliet et al., 2010; Dahlgren et al., 2023; Stringer et al., 2024). In intensive hematological treatment, mucosal barrier injury is a recognized pathological state with infectious and inflammatory consequences (Blijlevens et al., 2000). In breast cancer chemotherapy, lower baseline microbial alpha diversity predicts greater gastrointestinal symptoms and greater chemotherapy-induced microbiome disruption, supporting the relevance of pretreatment mucosal and microbial risk states (Otto-Dobos et al., 2024). In CRF, the same pathology can be viewed not only as gastrointestinal toxicity but also as sensory amplification. When tight junctions, mucus, and crypt renewal are intact, luminal organisms and their products remain spatially separated from lamina propria sensors. When therapy disrupts that separation, the same products can be read as danger or metabolic stress by macrophages, dendritic cells, enteroendocrine cells, and sensory neurons. The signal that reaches the brain may therefore reflect epithelial alarm, microbial exposure, and neural transduction rather than microbial abundance alone (Yonekura et al., 2022). This view also explains why gastrointestinal symptoms and fatigue cluster without being identical: diarrhea may reflect epithelial transport and motility, whereas fatigue may reflect immune–metabolic signaling and central interoceptive gain. In this framework, mucosal injury is not only a gastrointestinal toxicity. It is a molecular exposure state in which luminal metabolites and microbial products become available to epithelial, immune, endocrine and neural sensors.

The gut is also affected by tumor-host neural and endocrine output. Cancer-induced β-adrenergic ileopathy can promote dysbiosis independently of direct chemotherapy exposure, arguing against a one-way model in which microbes simply act on the brain. Stress, sleep disruption, tumor burden, and autonomic tone can alter the intestinal niche, and the altered gut can then feedback to immune and neural systems. A clinically useful model must therefore ask whether a reversible mucosal state is present and still responsive to intervention. The evidence gradient across these domains is uneven. Direct CRF evidence is strongest for associations with microbiome features and kynurenine-inflammatory signals and moderate for mucositis-linked inflammatory and metabolic plausibility. Causal evidence for vagal, NTS, or DRN pathways in CRF remains largely absent. Recent plasma metabolomic profiling of CRF highlighted tryptophan metabolism among discriminating pathways, while underscoring the need to separate fatigue-related signals from cachexia and chemotherapy-related metabolic effects (Okinaka et al., 2024).

## 5 Medicinal plant-derived bioactive compounds and gut mediator–sensor modules

Medicinal plant-derived bioactive compounds enter this framework through their interactions with the gut microbiota, which include ecological remodeling of microbial communities and microbiota-dependent biotransformation; some constituents may also act directly on host targets. Polyphenols, polysaccharides, saponins, and alkaloids are often poorly absorbed in the upper gut and reach the colon, where resident microbes convert them into metabolites that overlap with the mediators discussed below, among them SCFAs, indole derivatives, and secondary bile acids. Saponins are triterpenoid glycosides, so this group is also the terpenoid-derived class represented in Table 2. The pharmacological action of a plant-derived compound in CRF therefore depends on the same variables that govern any gut-derived signal: the identity and dose of the parent compound, the metabolic capacity of the recipient’s microbiota, the integrity of the epithelial barrier, and the sensor engaged by the resulting metabolite. Reviews of traditional Chinese medicines organized by their effects on the gut microbiota make the same point, describing how SCFAs, bile acids, 5-hydroxytryptamine, and other microbial products carry the host response (Su et al., 2024), and phytochemical reviews in oncology list gut microbiota among the mechanisms through which plant compounds act (Zhao et al., 2025). These observations shift the unit of analysis from the plant compound alone to the plant–microbiota–host axis: the same parent molecule can produce different biological outcomes across individuals because the microbial conversion step differs. This substrate-to-mediator logic is applied to each module below.

**Table 2:** Plant-derived inputs and their microbial transformation into host-sensed mediators.

Plant class	Representative parent compound	Microbial transformation/ecological effect	Microbially generated product/representative host pathway	CRF evidence level
Polyphenols	Ellagic acid, daidzein, quercetin	Deglycosylation, ring fission, dehydroxylation; prebiotic and antimicrobial modulation	Urolithins, equol, small phenolic acids (targets vary by metabolite; e.g., equol at ERβ)	Mechanistic exemplar; no direct CRF evidence
Saponins	Ginsenosides Rb1, Rb2, Rc	Stepwise gut microbial deglycosylation	Compound K (absorbed aglycone)	Mechanistic exemplar
Alkaloids	Berberine	Nitroreductase reduction; antimicrobial remodeling	Dihydroberberine → berberine (microbiota-gated absorption)	Mechanistic exemplar
Polysaccharides	Astragalus, ginseng polysaccharides	CAZyme-mediated fermentation; cross-feeding	SCFAs → FFAR2/FFAR3, HDAC, vagal afferents	Plausible; indirect CRF evidence

In this table summarizes representative plant-derived compound classes and their microbial transformation. The listed examples are mechanistic exemplars; direct evidence for cancer-related fatigue is limited and is addressed separately in the text. ERβ, estrogen receptor beta; CAZyme, carbohydrate-active enzyme.

### 5.1 Medicinal plant-derived compounds as modulators of gut microbial community structure and function

Plant-derived compounds act on the gut microbiota in two directions. The first is ecological: plant constituents change which taxa proliferate and which metabolic functions the community expresses. The second, microbial biotransformation of the parent compound, is treated in the next subsection. This effect should be read in functional rather than purely compositional terms, because a compound can shift community output—SCFAs, indoles, and bile-acid profiles—without a reproducible change in the names of the dominant taxa (Cardona et al., 2013).

The routes are several and often concurrent. Non-digestible polysaccharides and many polyphenols act as growth substrates for specific taxa, so that primary degraders cross-feed secondary fermenters, including butyrate producers (Su et al., 2024; Cardona et al., 2013; Sonnenburg et al., 2010; Rakoff-Nahoum et al., 2016). Other compounds, exemplified by berberine and several polyphenols, are directly antimicrobial and suppress sensitive taxa while tolerant organisms expand (Cardona et al., 2013). Exposure to plant-derived substrates may favor microbial communities enriched in carbohydrate- and xenobiotic-metabolizing functions, including glycosidase activity (Cardona et al., 2013). Other microbial enzymes, such as β-glucuronidases and bile-salt hydrolases, may further modify enterohepatic or bile-acid-associated host exposure. The net effect is a shift in community function that feeds directly into the mediator modules discussed below.

The magnitude, and sometimes the direction, of this modulation depends on the recipient’s baseline microbiota, prior antibiotic exposure, and diet, so the same compound does not produce the same community shift in every individual (Su et al., 2024; Cardona et al., 2013). For CRF this matters in a specific way: a plant-derived intervention is better framed as a modulator of microbial function whose effect must be measured in the enrolled population, not assumed from taxonomy or from healthy-volunteer data. This functional framing, rather than a list of taxa, is what connects plant compounds to the mediator–sensor logic that follows.

### 5.2 Plant-derived bioactive compounds and their gut microbial biotransformation

Whether a plant-derived compound acts through the gut microbiota cannot be tested until the relevant microbial conversion has been named. Polyphenols, the largest class of medicinal plant constituents, are the clearest example. Most occur in planta as glycosides or esters that are poorly absorbed in the small intestine and reach the colon largely intact, where bacterial glycosidases remove the sugar moieties and a downstream sequence of ring fission, demethylation, and dehydroxylation converts the aglycones into small phenolic acids. Those phenolic acids, rather than the parent compounds, are the species recovered in plasma and urine (Selma et al., 2009). The microbial step, not the parent molecule, determines which compound the host is actually exposed to.

The same principle applies to the other major classes. Ellagitannins are hydrolyzed to ellagic acid and then dehydroxylated to urolithins by a restricted set of species; because the responsible taxa are unevenly distributed, individuals fall into urolithin metabotypes that fix how much of the active metabolite is generated (Tomás-Barberán et al., 2014). Isoflavones follow the same pattern: only part of the population harbors the bacteria that reduce daidzein to equol, so equol-producer status, not daidzein dose, governs exposure to the more potent estrogenic metabolite (Setchell and Clerici, 2010). Ginseng saponins are deglycosylated stepwise by gut bacteria to compound K, the aglycone that is absorbed and that accounts for most of the systemic exposure attributed to the major protopanaxadiol-type ginsenosides (Kim, 2018). Berberine is an alkaloid case in which the microbiota does not merely form an active metabolite but rescues a nearly unabsorbable molecule: a reduction to dihydroberberine by bacterial nitroreductases is one established, microbiota-dependent route by which this otherwise poorly absorbed alkaloid reaches the circulation, where it is re-oxidized to berberine after uptake (Feng et al., 2015). These transformations are well documented *in vitro* and in human pharmacokinetic studies, but they are mechanistic exemplars from outside oncology; none of them yet constitutes direct evidence that a plant-derived compound improves CRF.

Plant polysaccharides occupy the opposite extreme of the same logic. Most intact plant polysaccharides have limited direct absorption, and a substantial part of their activity depends on microbial fermentation, of which short-chain fatty acids are the most studied but not the only output; these then engage the epithelial, immune, and vagal receptors discussed in the sections that follow (Su et al., 2024). These examples lead to a single conclusion: the administered compound is a substrate, and its pharmacological identity in a given patient is substantially shaped by that patient’s microbiota. A metabotype, a producer status, or a measured microbial metabolite is therefore the appropriate pharmacodynamic readout for plant-derived interventions in CRF, rather than the parent dose alone. Whether a patient can form the active metabolite can be established before enrollment. Urolithin metabotypes are determined from urinary or plasma urolithin profiles after a standardized ellagitannin-rich challenge, and equol-producer status is determined from plasma equol after a daidzein challenge or from the gene clusters responsible for the conversion in stool metagenomes. In a CRF trial these assignments are most useful as pre-specified stratification variables at Stage 1 of the design described in Section 10, so that participants who can generate the active metabolite are analyzed separately from those who cannot. A negative result can then be attributed to a missing microbial conversion step rather than to an inactive parent compound.

### 5.3 SCFAs: local epithelial metabolism, immune tone, and vagal Chemosensing

Short-chain fatty acids (SCFAs) are often treated as broadly beneficial microbial metabolites, but their pharmacology is compartment-specific. Acetate, propionate, and butyrate differ in luminal concentration, receptor preference, tissue distribution, and cellular metabolism. Butyrate is consumed avidly by colonocytes and mainly acts as a local epithelial fuel and immune regulator; propionate and acetate have greater systemic availability but are less specific as neural signals. SCFAs signal through free fatty acid receptor 2 (FFAR2/GPR43) and free fatty acid receptor 3 (FFAR3/GPR41), and butyrate can also inhibit histone deacetylases (HDACs), influencing epithelial and immune transcriptional programs (Cryan et al., 2019; Mayer et al., 2022; Macpherson et al., 2023; Dalile et al., 2019; Koh et al., 2016). FFAR3/GPR41 has been linked to autonomic regulation, and experimental systems show that SCFAs can act on vagal afferents (Brown et al., 2003; Kimura et al., 2011; Onyszkiewicz et al., 2019; Goswami et al., 2018). For CRF, the issue is not whether butyrate is anti-inflammatory in general, but whether loss of fermentation compromises epithelial barrier function and changes gut-to-brain sensory input. Most proposed SCFA interventions lack pharmacodynamic proof: fecal butyrate alone is a weak endpoint because it reflects production, consumption, transit, diet, and sampling conditions. A credible CRF study should pair stool metagenomic function with fecal and plasma SCFAs, barrier markers, mucosal or systemic inflammatory indices, HRV, and a phenotype-specific fatigue measure.

SCFA effects are context-dependent, which makes rescue experiments especially useful. Butyrate can support epithelial metabolism and regulatory immune tone, but dose, location, and disease state matter: excessive luminal fermentation in a patient with diarrhea or mucositis may worsen bloating, pain, or intake. Acetate and propionate have systemic metabolic effects that are not equivalent to butyrate’s local epithelial actions. Restoring SCFAs is therefore a molecular question: which SCFA, in which compartment, at which treatment phase, and with which sensor as the intended target? In animal CRF models, antibiotics or chemotherapy could be used to create a low-fermentation state, followed by butyrate, tributyrin, or a defined fermenting consortium. The design is most informative when combined with vagal interruption or receptor-level manipulation where feasible, together with mucosal cytokines, epithelial markers, NTS activation, and activity behavior. If butyrate restores barrier integrity without changing fatigue-like behavior, the pathway may be mucosal but not fatigue-relevant; if behavior changes without barrier or vagal markers, another mechanism becomes more likely. The key point is not that SCFAs are generally anti-inflammatory, but that they provide a testable molecule–sensor axis involving FFAR2/FFAR3, hydroxycarboxylic acid receptor 2 (HCAR2), HDAC-dependent transcription and epithelial–immune metabolic coupling.

The SCFA hypothesis is therefore biologically plausible, but it remains incompletely tested in CRF. Current support comes mainly from non-cancer fatigue models, general SCFA biology in epithelial and immune regulation, and experimental work on vagal afferents. A proof-of-concept exists outside oncology: the traditional formula Jiaotaiwan raised serum SCFAs and engaged a cAMP-PKA-CREB-BDNF pathway in parallel with antidepressant effects in a multicenter randomized study (Huang et al., 2025), showing that a plant-derived multi-component intervention can move a defined SCFA readout, although CRF-specific replication is lacking. The endpoint of that trial was antidepressant response, so the result shows that a plant-derived multi-component intervention can shift a defined SCFA readout in a depressed population; whether a comparable shift improves the physical or motivational domains of CRF listed in Table 1 has not been tested. Most human CRF microbiome studies do not measure taxonomy, SCFA concentrations, barrier markers, and autonomic readouts in the same patients. A more decisive test would require an SCFA-directed intervention to change a prespecified pharmacodynamic biomarker and a matched fatigue domain in an enriched CRF cohort. Until that experiment is performed, the SCFA model should be treated as a strong mechanistic hypothesis rather than an established CRF pathway. Fecal SCFA concentration alone is not a stable proxy for target engagement at the receptor or tissue level.

### 5.4 Tryptophan metabolism: serotonin, kynurenine, and indoles must be separated

Tryptophan metabolism is useful precisely because it links microbial chemistry, host immunity, enteroendocrine signaling, and monoamine biology. It is also easy to overinterpret. Tryptophan is not simply a serotonin precursor. Host pathways convert it into serotonin through tryptophan hydroxylase, into kynurenines through indoleamine 2,3-dioxygenase 1/2 (IDO1/IDO2) and tryptophan 2,3-dioxygenase (TDO), and into downstream compounds through kynurenine 3-monooxygenase (KMO), kynurenine aminotransferase (KAT), and related enzymes. Microbial pathways generate indole, indole-3-aldehyde, indole-3-acetic acid, tryptamine-related compounds, and other derivatives. Formula-based work reinforces the microbiota dependence of this chemistry: the antidepressant effect of Zhi-zi-chi Decoction was attributed to *Lactobacillus rhamnosus* and gut-microbiota metabolism rather than to the parent formula alone (He et al., 2025). These branches differ in tissue location, receptor biology, and pharmacological meaning (Yano et al., 2015; Ye et al., 2021; Zelante et al., 2013; Agus et al., 2018; Platten et al., 2019). Recent cancer-focused reviews likewise separate kynurenine, serotonin, and microbial indole branches when discussing tumor immunity, nutrition, cachexia, and neuroactive signaling (Molfino et al., 2024; Yan et al., 2024). The kynurenine branch has the strongest clinical symptom evidence. Inflammation-induced tryptophan breakdown has been related to anemia, fatigue, and depression in cancer, and longitudinal data in colorectal cancer survivors link tryptophan and kynurenine-pathway metabolites with fatigue and quality of life (Lanser et al., 2020; Holthuijsen et al., 2024). Breast cancer survivor data also link tryptophan-kynurenine metabolites with psychoneurological symptom clusters (Li et al., 2022). These findings make the kynurenine-to-tryptophan ratio (Kyn: Trp) and downstream kynurenines reasonable enrichment variables, but they do not report DRN serotonin function.

This distinction matters therapeutically. IDO1 inhibitors, TDO inhibitors, KMO modulators, and anti-inflammatory strategies are not interchangeable. IDO1 may be induced in immune and tumor-associated contexts; TDO may be liver- or tumor-driven; and KMO and KAT shape the balance between neuroactive and potentially neuroprotective kynurenines. A high Kyn: Trp ratio can identify pathway activation, but it does not identify the optimal pharmacological node. In oncology, manipulation of tryptophan metabolism also has to be considered alongside tumor immunity and immunotherapy interactions. Microbial indole biology requires the same cell-specific caution. Aryl hydrocarbon receptor (AHR) activation can be protective or pathogenic depending on ligand, cell type, exposure duration, and tissue context. Epithelial AHR–interleukin-22 (IL-22) signaling may support barrier defense, whereas AHR effects in other immune or tumor compartments may be undesirable. In a formula or probiotic study, a shift in an indole metabolite is not sufficient; the relevant AHR-responsive program must be engaged in the target tissue and linked to barrier repair or fatigue-domain improvement. Untargeted metabolomics alone cannot resolve this. Low-abundance indole aldehydes and related isomers require targeted liquid chromatography–tandem mass spectrometry (LC–MS/MS), stable annotation, and paired stool-blood sampling before concluding that a probiotic or dietary substrate restores indole-AHR signaling. Host kynurenine metabolism, enterochromaffin-cell serotonin biology and microbial indole-AHR signaling are related branches of tryptophan metabolism, but they do not report the same molecular event.

The kynurenine branch is therefore best viewed as an IDO/TDO/KMO/KAT molecular pathway whose net output, rather than any single metabolite, may interact with central serotonergic and dopaminergic systems. Among tryptophan-derived pathways, it currently offers the strongest translational rationale, but the direction of any kynurenine effect on DRN serotonin function in CRF remains unresolved. Peripheral Kyn: Trp ratios are useful for identifying pathway activation, yet they cannot guide the choice among IDO1-, TDO-, or KMO-directed strategies. Where a downstream metabolite panel is available, an elevated QA/KA ratio would suggest preferential flux toward the HK/QA branch relative to the KA branch, whereas lower HK and QA accompanied by increased KA would be more consistent with reduced KMO-dependent flux. These metabolite patterns indicate pathway direction but do not, by themselves, identify the enzyme that should be targeted. These enzymes intersect with tumor immunity and therefore require careful evaluation. For microbial indoles, the key unresolved question is not whether AHR activation occurs, but whether it occurs in the right cell type, at the right magnitude, and with the right transcriptional consequences to strengthen barrier defense and reduce fatigue. Answering that question requires targeted LC–MS/MS, tissue-level demonstration of AHR-responsive programs, and evidence that the relevant endpoints are mediated through this pathway rather than through parallel mechanisms.

### 5.5 MAMPs, bile acids, and gut sensory-cell signaling

Microbe-associated molecular patterns (MAMPs) provide a more direct inflammatory signal. LPS, peptidoglycan, flagellin, and bacterial DNA can activate mucosal immune cells when barrier selectivity is impaired. The pharmacological conclusion is not necessarily to block Toll-like receptor 4 (TLR4) systemically—in oncology patients, broad innate-immune blockade may increase infection risk and interfere with tumor immunity. A safer strategy is to reduce mucosal exposure by repairing the epithelial gate, improving mucus and tight-junction function, or shifting the microbial ecology that drives local inflammatory tone. Bile acid signaling is less well characterized in CRF, but it is relevant to fatigue phenotypes that include appetite disturbance, nausea, or gastrointestinal symptoms. Gut bacteria convert primary to secondary bile acids, which signal through farnesoid X receptor (FXR) and Takeda G protein-coupled receptor 5 (TGR5) and influence metabolism, inflammation, enteroendocrine secretion, and vagal pathways (Kaelberer et al., 2018; Buchanan et al., 2022; Wu et al., 2020; Wahlström et al., 2016). Cancer therapy, antibiotics, dietary change, and cholestasis can remodel bile-acid pools. In a fatigue context, bile acids may act indirectly through appetite, nausea, diarrhea, energy balance, and autonomic signaling.

Enteroendocrine and neuropod-cell biology provides the cellular bridge between luminal chemistry and neural activity. Enterochromaffin cells (ECs) release 5-hydroxytryptamine (5-HT) onto 5-HT3 receptor-expressing vagal afferents, whereas neuropod cells form synaptic-like glutamatergic contacts with vagal terminals. These routes transmit information faster than endocrine diffusion and may explain why gut perturbation can rapidly alter malaise or feeding behavior. For CRF pharmacology, a stool-metabolite change is interpretable as a gut–brain effect only if it changes what mucosal sensory cells detect or what vagal/humoral routes transmit. The 5-HT3 interface shows why phenotype control is essential. 5-HT3 antagonists are standard antiemetics in chemotherapy care; if fatigue improves after a 5-HT3-related intervention, the effect may be secondary to reduced nausea, better intake, improved sleep, or less anticipatory distress. Bile acids raise a similar interpretive problem because they influence gut hormone release, motility, appetite, and metabolic signaling. These mechanisms can be separated only when targeted bile-acid panels are paired with glucagon-like peptide-1 (GLP-1), peptide YY (PYY), or cholecystokinin (CCK) readouts, gut symptoms, and fatigue-domain outcomes.

Overall, MAMPs, bile acids, and enteroendocrine/neuropod-cell signaling should be interpreted as plausible but incompletely validated mediator–sensor modules in CRF. MAMP sensing through TLR4 and related pattern-recognition receptors is well established in mucosal immunology, bile-acid signaling through FXR and TGR5 is pharmacologically well characterized, and enteroendocrine or neuropod-cell pathways provide credible routes for rapid gut-to-brain communication. Direct fatigue-specific evidence remains limited for these modules, particularly for causal transmission through vagal or humoral pathways in cancer and treatment-related fatigue models. These mechanisms are therefore best treated as testable candidates rather than established CRF pathways. Table 3 summarizes their biological sources, evidence status, candidate interventions, and pharmacodynamic readouts. The spatial organization of these mediator–sensor modules at the injured mucosal interface is summarized in Figure 2.

**Table 3:** Gut-derived mediator classes, host sensors, and molecular interpretation in CRF.

Mediator/process	Primary biological source and host sensor	Evidence status for CRF	Candidate interventions	Essential pharmacodynamic readouts	Representative supporting evidence
SCFA loss and fermentation failure	Anaerobic fermentation by fiber-utilizing taxa; FFAR2/FFAR3, HCAR2 and HDAC-linked effects in epithelium, immune cells and sensory pathways.	Direct CRF evidence remains mostly associative; ME/CFS multi-omics provide stronger functional evidence for reduced butyrate-producing capacity.	Fermentable fiber, prebiotics, defined consortia, butyrate/tributyrin, diet-formula interventions designed around fermentation capacity.	Stool metagenomic function, fecal and plasma SCFAs, barrier markers, mucosal/systemic cytokines, HRV and physical-fatigue endpoints.	Guo et al. (2023), Xiong et al. (2023), Dalile et al. (2019), and Goswami et al. (2018)
Tryptophan-kynurenine activation	Host IDO1/IDO2/TDO and downstream KMO/KAT branches in inflammatory cells, liver, tumor and mucosa.	Clinical studies relate Kyn: Trp and kynurenines to fatigue, depression, anemia and HRQoL in cancer populations.	Anti-inflammatory strategies, exercise/nutrition, carefully justified kynurenine-axis modulation, subgroup-enriched trials.	Trp, Kyn, Kyn: Trp, KA, QA, HK and related ratios; CRP/IL-6; anemia and depression covariates.	Okinaka et al. (2024), Molfino et al. (2024), Yan et al. (2024), Lanser et al. (2020), Holthuijsen et al., 2024; Li et al. (2022), and Holthuijsen et al. (2025)
Microbial indoles and AHR biology	Microbial tryptophan catabolism; AHR signaling in epithelium and immune cells; enteroendocrine sensing of bacterial catabolites.	Strong mucosal-immunology rationale; direct CRF data are absent or preliminary and are best treated as hypothesis-generating.	Dietary substrates, microbiota restoration, formula studies with targeted metabolomics and cell-type readouts.	Targeted indole panel, AHR target genes, IL-22 pathway markers, epithelial integrity and paired stool-blood data.	Ye et al. (2021), Zelante et al. (2013), Agus et al. (2018), and Platten et al. (2019)
MAMP exposure after barrier injury	Barrier-dependent access of LPS, peptidoglycan, flagellin and bacterial DNA to macrophages, dendritic cells and sensory terminals.	Strong mucositis and sickness-behavior biology; CRF-specific causality incompletely tested.	Barrier repair, mucus/tight-junction support, local anti-inflammatory strategies, microbiome remodeling rather than broad systemic TLR blockade.	Plasma LBP/sCD14, endotoxin-related assays with caution, tight-junction proteins, permeability assays, mucositis severity.	Sonis (2004), Dahlgren et al. (2023), Yonekura et al. (2022), and Tracey (2007)
Bile-acid remodeling	Microbial bile-salt hydrolase and dehydroxylation activity; FXR/TGR5, enteroendocrine and vagal interfaces.	Plausible for appetite, nausea and energy-balance phenotypes; direct CRF evidence is minimal and phenotype-dependent.	Dietary modulation, bile-acid sequestration or receptor modulation only in selected contexts.	Targeted bile-acid panel, GLP-1/PYY/CCK, appetite and nausea measures, gut symptom stratification.	Wahlström et al. (2016) and Chiang and Ferrell (2020)
Enterochromaffin/neuropod-cell signaling	Enterochromaffin-cell 5-HT and glutamatergic neuropod-vagal synapses responding to nutrients and microbial metabolites.	Strong basic biology; CRF evidence remains indirect and requires route-specific testing.	5-HT3-related gut-sensory strategies, antiemetic-phenotype studies, mucosal metabolite modulation.	Nausea and fatigue measured separately; vagal/NTS readouts in models; EC markers where feasible.	Goswami et al. (2018), Yano et al. (2015), Kaelberer et al. (2018), Bellono et al. (2017))

In this table indicate representative mechanistic and translational evidence supporting each mediator–sensor module. Direct CRF evidence remains uneven: it is strongest for microbiome and kynurenine associations, weaker for bile-acid and enteroendocrine-vagal mechanisms, and largely preclinical for route-specific causality. ME/CFS, myalgic encephalomyelitis/chronic fatigue syndrome; Trp, tryptophan; Kyn, kynurenine; KA, kynurenic acid; QA, quinolinic acid; HK, 3-hydroxykynurenine; CRP, C-reactive protein; IL-6, interleukin-6; LBP, lipopolysaccharide-binding protein; sCD14, soluble CD14; HRQoL, health-related quality of life.

**Figure 2:**
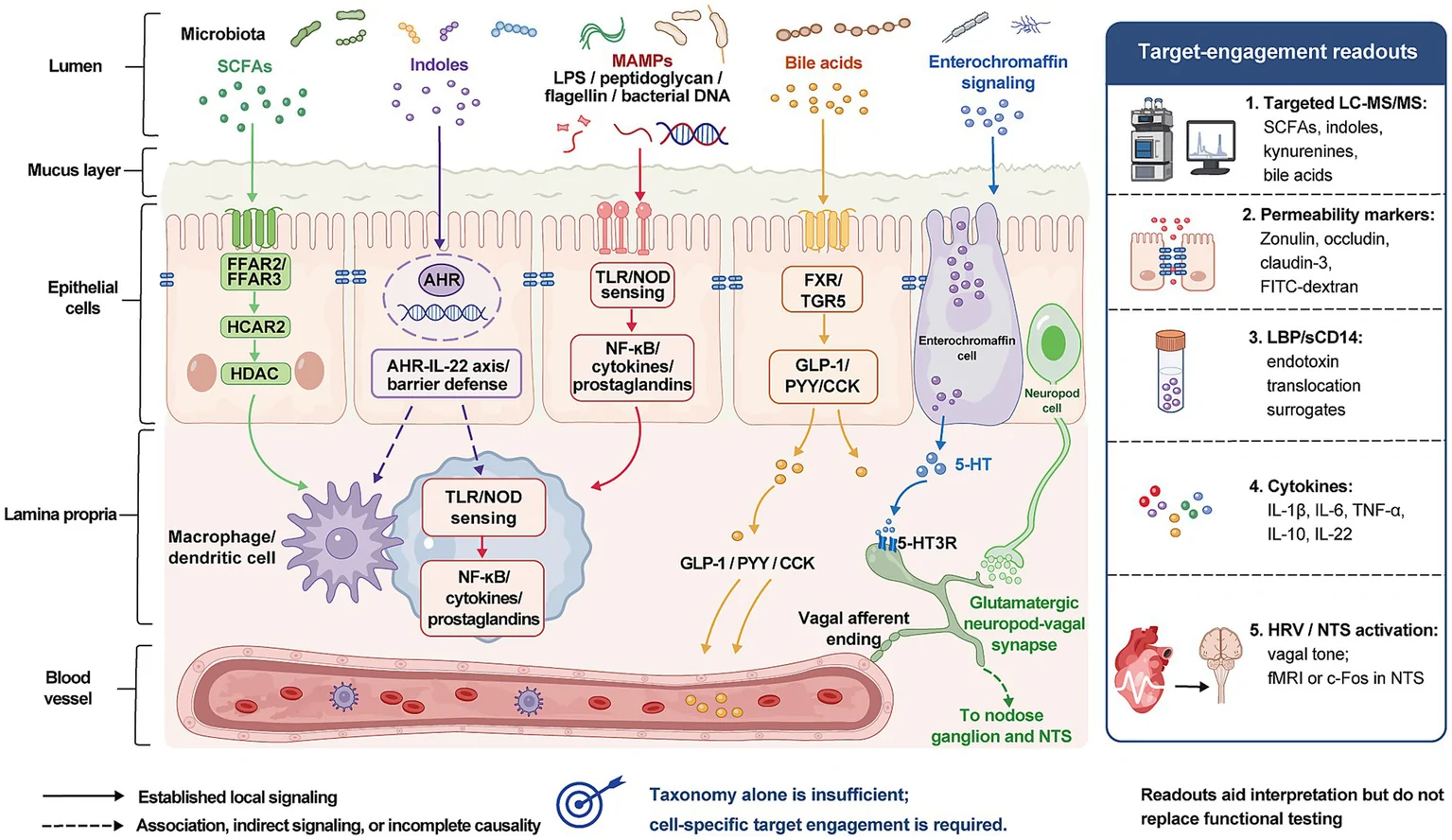
Mucosal mediator–sensor architecture. Treatment-injured intestinal mucosa is shown as a spatial interface in which microbial metabolites, MAMPs, bile acids, enterochromaffin-cell serotonin, and neuropod-cell synapses are detected by epithelial, immune, endocrine, and neural sensors. Taxonomic association alone is insufficient for molecular interpretation. Mechanistic inference requires cell-specific biological engagement, compartment-aware mediator measurement, and matched readouts of barrier, inflammatory, autonomic, and NTS-related activity. Plant-derived substrates are metabolized at this interface into the microbial mediators and host signals shown.

## 6 Routes from the gut to the nervous system: route dominance, not route exclusivity

The vagus nerve is the most direct anatomical route from the gut to the brainstem. Subdiaphragmatic vagal afferents terminate in the NTS, and their peripheral endings can detect mechanical, nutrient, hormonal, and immune cues near the mucosal interface. Classic immune-to-brain studies show that vagotomy can attenuate selected behavioral responses to IL-1β and LPS, and vagal afferent activity can encode inflammatory features (Maier et al., 1998; Ek et al., 1998; Steinberg et al., 2016). The efferent inflammatory reflex provides a second limb through which vagal activity restrains cytokine production via α7 nicotinic mechanisms (Pavlov and Tracey, 2012). These findings make vagal modulation plausible for CRF, but not universal. The area postrema and the adjacent dorsal vagal complex provide a parallel humoral interface: circulating mediators can influence brainstem processing there without requiring broad penetration of the blood–brain barrier. This route is especially relevant in advanced cancer, where systemic inflammation, tumor-derived mediators, liver metabolism, and cachexia can generate humoral signals that may bypass or accompany vagal input. A vagotomy-insensitive fatigue phenotype may therefore be humoral, metabolic, or central-plasticity dominant rather than unexplained (Breit et al., 2018; Bonaz et al., 2018; Ran et al., 2022; Holt, 2022).

Immune-cell routes may become more important in chronic disease. Gut-conditioned immune cells, low-grade systemic inflammation, and altered meningeal or barrier immune tone could influence central nervous system (CNS) networks over longer time scales. These pathways are slower than vagal sensory transmission and may not explain acute mucositis-driven fatigue, but they may matter in survivorship, where fatigue can persist after treatment ends. Route dominance is therefore more useful than route exclusivity. In acute treatment-associated CRF with mucositis, diarrhea, or abdominal radiotherapy exposure, vagal and area postrema/NTS routes may carry much of the signal. In advanced cancer with cachexia, systemic cytokines, liver metabolism, anemia, and muscle catabolism may dominate. In post-treatment survivorship, central learning, sleep disruption, deconditioning, and persistent autonomic change may maintain fatigue after gut injury has improved. Microbiota–gut–brain reviews support this multi-route architecture, in which neural, immune, endocrine, and metabolic channels operate in parallel (Cryan et al., 2019).

The metabolites formed from plant-derived substrates travel the same vagal and humoral routes, so the route-dominance logic applies to plant-derived interventions as well. Experimental design follows from route dominance. If microbiome manipulation improves fatigue-like behavior in mice, vagotomy or selective vagal silencing can test whether the effect is vagal. If the effect persists after vagal interruption, area postrema signaling, circulating metabolites, immune cells, food intake, and tumor burden need evaluation. Conversely, improvement after transcutaneous auricular vagus nerve stimulation (taVNS) without HRV, inflammatory, or brainstem engagement should not be interpreted as normalization of gut-derived afferent signaling; arousal, sleep, mood, expectation, or nonspecific autonomic effects remain possible.

## 7 Brainstem and monoaminergic convergence nodes: evidence and limitations

### 7.1 NTS, dorsal vagal complex, and interoceptive gain

The NTS is the first central station for most vagal afferent input and lies within the dorsal vagal complex beside the area postrema. It is not a passive relay. Brainstem mapping studies describe molecularly distinct NTS neuronal populations that project to the parabrachial nucleus, locus coeruleus, hypothalamus, medullary autonomic centers, and midbrain structures. This organization makes the NTS an appropriate convergence site to measure in CRF models because it can integrate vagal and humoral signals and distribute them to circuits controlling arousal, malaise, autonomic tone, and activity. Inflammatory challenge activates caudal brainstem catecholamine pathways, and lesions of ascending catecholaminergic neurons can alter sickness-related locomotor suppression in experimental models (Gaykema and Goehler, 2011). Direct evidence that NTS activity causes CRF in validated cancer models or patients is still sparse. The NTS is better treated as a measurable convergence site than as a proven clinical target. In animals, useful readouts include NTS c-Fos mapping, single-nucleus transcriptomics, fiber photometry, vagal recording, and projection-specific manipulation. In patients, brainstem-optimized fMRI, HRV, and inflammatory-metabolic biomarkers can test engagement if spatial resolution and physiological noise are handled carefully.

Projection anatomy also weighs against a narrow NTS-DRN account. The NTS projects to the parabrachial nucleus, which participates in visceral malaise and aversive bodily state; to the locus coeruleus, which regulates arousal, vigilance, and effort allocation; and to hypothalamic nuclei, which couple interoception to endocrine and energy-balance responses. These circuits can generate fatigue-like output without a direct serotonergic mechanism. In some phenotypes, NTS-LC or NTS-PBN signaling may be more relevant than NTS-DRN signaling. This broader brainstem network complicates biomarker selection. HRV indexes autonomic regulation but cannot localize a signal to the NTS, vagal afferents, or the efferent inflammatory reflex. Brainstem fMRI provides regional information but is technically difficult because the nuclei are small and the signal is affected by motion and physiological noise. Plasma cytokines and metabolites are accessible but indirect. The strongest design is one in which gut engagement, autonomic or humoral readouts, brainstem activity, and fatigue-domain improvement change in a coherent temporal sequence. NTS and DRN readouts should be treated as convergence and engagement markers unless route-specific manipulation shows that they are required for the fatigue phenotype.

### 7.2 DRN serotonin: fatigue-relevant but compartment-specific

The DRN matters because serotonin regulates arousal, movement, waiting, aversion, sleep–wake state, and perceived effort. It is also a well-characterized neurotransmitter system with established pharmacological tools. However, DRN serotonin neurons are heterogeneous in molecular identity, projection pattern, and behavioral effect (Pollak Dorocic et al., 2014; Herbert, 1992; Ren et al., 2019; Huang et al., 2019). This heterogeneity helps explain why global serotonergic manipulation has not produced a specific and reproducible CRF treatment: serotonin in one compartment may facilitate motor readiness, whereas spillover in another can suppress firing. Central fatigue physiology provides a useful example: serotonin spillover onto the axon initial segment of spinal motoneurons can activate 5-HT1A receptors and inhibit action potential initiation during sustained motor activity (Cotel et al., 2013). Peripheral cytokines and LPS can also alter serotonin turnover or extracellular serotonin in the DRN, with evidence for vagal and prostaglandin involvement in inflammatory models (Clement et al., 1997; Hollis et al., 2006). These data support plausibility for gut-immune effects on serotonergic fatigue biology, but they do not mean that peripheral tryptophan or Kyn: Trp reports DRN serotonin function.

The NTS-to-DRN connection remains at an early discovery stage. Anatomical tracing and input mapping support medullary input to raphe serotonin systems, but transmitter phenotype, postsynaptic target, synaptic polarity, and relevance to chemotherapy- or tumor-associated fatigue remain unresolved. Optogenetic and imaging studies show that DRN serotonin activation can produce different brain-wide and behavioral states depending on timing and context (Correia et al., 2017; Hamada et al., 2024). Causal testing would require retrograde tracing from DRN, single-cell profiling of DRN-projecting NTS neurons, slice electrophysiology, chemogenetic inhibition during fatigue-like behavior, and parallel measurement of gut biological engagement. Adjacent networks should be considered at the same time. The parabrachial nucleus translates visceral malaise into affective and arousal states; the locus coeruleus controls alertness and effort allocation; hypothalamic projections link interoception to endocrine and energy-balance responses; and midbrain dopamine systems regulate motivation and effort. Inflammation can alter motivation and motor activity through dopamine-related mechanisms as well as serotonin (Miller and Raison, 2016; Felger and Treadway, 2017; Capuron and Miller, 2011; Dinan and Cryan, 2017). A serotonin-only model is too narrow.

These circuit features have direct pharmacological consequences. Selective serotonin reuptake inhibitors (SSRIs), serotonin-norepinephrine reuptake inhibitors (SNRIs), and other global serotonergic drugs should not be treated as pathway-directed CRF therapies simply because the DRN influences behavioral state. Serotonergic tone can affect fatigue, mood, sleep, pain, and nausea in different directions depending on receptor subtype and circuit compartment. A patient with comorbid depression may benefit from antidepressant treatment, but that is different from correcting gut-derived serotonergic fatigue biology. Mechanistic studies need to distinguish treatment of comorbidity from target-directed anti-fatigue pharmacology. NTS and DRN readouts are most useful as pharmacodynamic markers in preclinical work. The strongest pathway evidence would require peripheral engagement, such as normalized SCFAs or barrier markers, to occur with reduced NTS activation and normalized DRN dynamics during activity tasks. Peripheral engagement without central change suggests that the gut signal may not be behaviorally relevant. Central change without peripheral engagement points toward a central or nonspecific mechanism. Both dissociations are informative, and both are necessary for the field to move beyond correlation. Plant-derived interventions face the same requirement: peripheral engagement by the microbial metabolite should be demonstrated before NTS or DRN readouts are interpreted as target engagement. The route-dominance model and candidate brainstem convergence nodes are shown in Figure 3.

**Figure 3:**
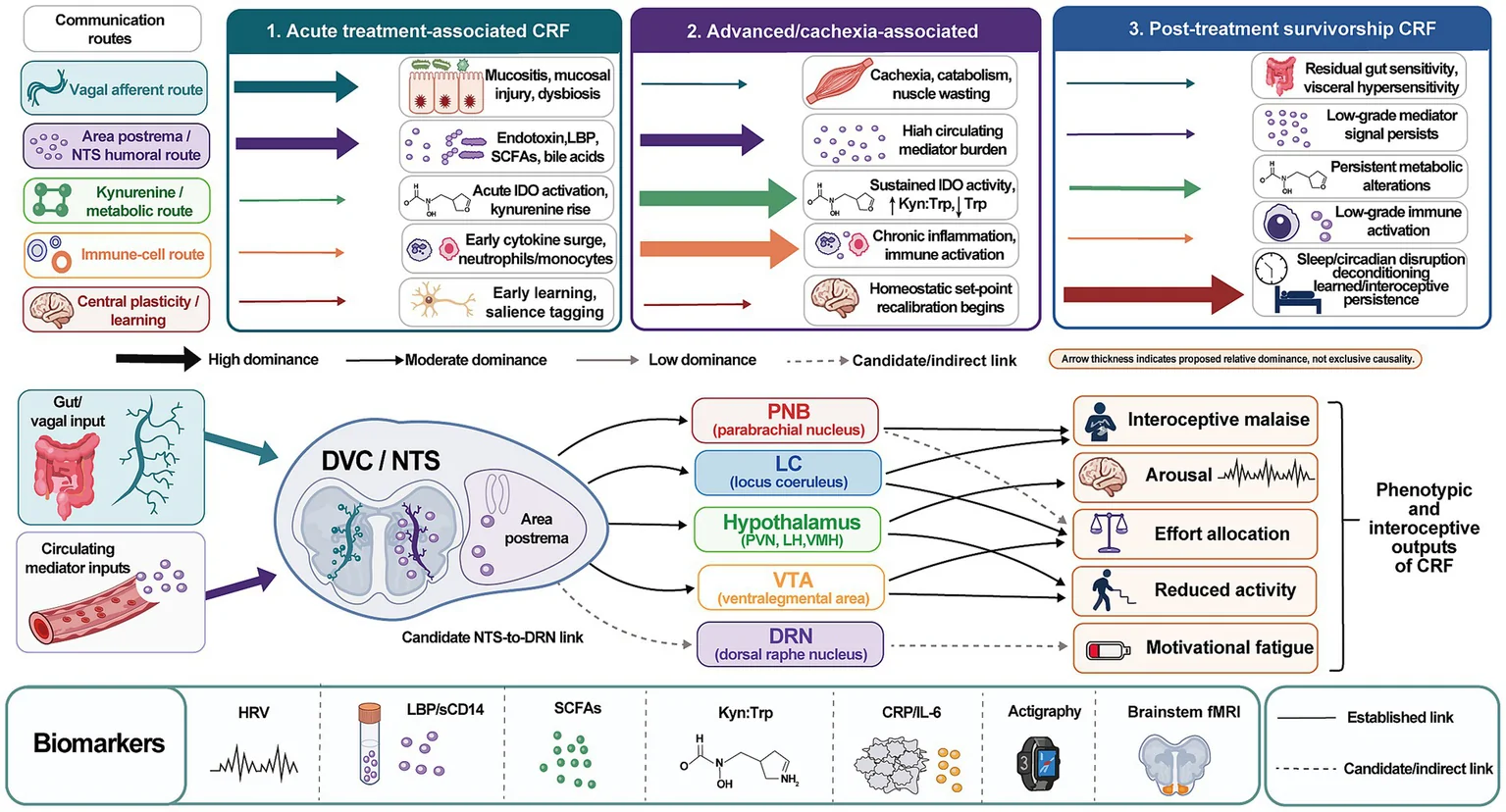
Route dominance and brainstem interoceptive networks in CRF. Disease stage may change the relative contribution of vagal, humoral, kynurenine/metabolic, immune-cell, and central-learning routes. Gut/vagal and circulating inputs converge on the dorsal vagal complex (DVC)/NTS and area postrema, then distribute to parabrachial nucleus (PBN), locus coeruleus (LC), hypothalamic, ventral tegmental area (VTA), and DRN nodes that regulate malaise, arousal, effort allocation, activity, and motivational fatigue. Arrow thickness indicates the route judged dominant in each setting rather than a validated effect size; dashed arrows indicate candidate or indirect links. Metabolites generated from plant-derived substrates traverse these same vagal and humoral routes.

## 8 Molecular target classes and validation logic

The mediator–sensor framework points to several molecular target classes. These include the SCFA/FFAR/HDAC axis, kynurenine-pathway enzymes, TLR4 and related pattern-recognition receptors, FXR/TGR5, 5-HT3 receptors, and vagal afferent signaling. Medicinal plant-derived bioactive compounds occupy the upstream end of this logic and can act through several routes, including supplying substrates for microbial biotransformation, remodeling the microbial community, and engaging host targets directly; when the intended mechanism is microbial biotransformation, validation depends on demonstrating the conversion step and the resulting mediator rather than the parent compound alone. Their translational maturity differs substantially. A target should not be considered pathway-relevant solely because an intervention changes fatigue scores. The stronger claim requires molecular engagement in the intended compartment, followed by a coherent change in the matched fatigue domain.

Barrier restoration and microbiome-directed interventions are closest to supportive-care testing. SCFA/FFAR/HDAC biology is plausible, but it requires evidence that the intervention changes host exposure rather than stool composition alone. Kynurenine-pathway strategies require caution because IDO/TDO biology intersects with tumor immunity. Enterochromaffin-cell 5-HT3 signaling is accessible clinically but strongly confounded by nausea and antiemetic effects. Vagal modulation is feasible, but it requires autonomic and brainstem engagement readouts. Central serotonergic approaches remain at the discovery stage for CRF unless they are tested in a defined biomarker subgroup or used for a clear comorbid indication. Barrier-directed pharmacology is the most upstream strategy because it could reduce exposure to microbial products, inflammatory cytokines, enteroendocrine perturbation, and vagal sensory activation. The term barrier repair should nevertheless be used precisely. A barrier trial should specify whether the intended effect is improved tight junctions, mucus, crypt regeneration, reduced microbial translocation, or lower mucosal cytokine signaling. Plasma LBP or soluble CD14 would support reduced microbial translocation, whereas preserved occludin or ZO-1 in a model would support tight-junction protection. These readouts are not interchangeable.

Microbiome-directed pharmacology needs the same functional specificity. The issue is not whether a probiotic is generally anti-inflammatory, but whether the organism or consortium supplies a missing function in the enrolled patient. A Lactobacillus-dominant product, a butyrate-producing consortium, a high-fiber diet, and fecal microbiota transplantation (FMT) are different interventions; grouping them as microbiome therapy hides the intended functional correction. This is especially important in oncology, where infection risk, immunotherapy interactions, and antibiotic exposure can change the risk–benefit balance (Routy et al., 2018; Davar et al., 2021; Baruch et al., 2021; Spencer et al., 2021; Wastyk et al., 2021; Khazaei et al., 2023; Belloni et al., 2024). Diet, antibiotics, probiotics, and FMT can influence cancer treatment response in selected contexts (Böhm et al., 2025), so supportive-care use should not rest on general anti-inflammatory claims. Oncology trials of synbiotics or probiotics have reported effects on treatment-related symptoms, and one systematic review suggests possible but limited evidence for CRF management. These data justify better-phenotyped mechanistic trials, not guideline-level pathway claims. For kynurenine biology, symptom-cluster studies support IDO-related mechanisms, but clinical translation should begin with measurement and subgrouping rather than drug blockade (Kim et al., 2023).

Neuromodulation fits the same target-class logic. taVNS is feasible, but stimulation parameters, auricular site, laterality, sham control, adherence, and autonomic state all affect interpretation. A fatigue response without HRV or inflammatory engagement would remain clinically interesting but would not validate a gut-vagus mechanism. Improved HRV without fatigue benefit would show physiological engagement without clinical translation; this distinction matters because HRV-related endpoints and HRV biofeedback have been studied in cancer populations but are not themselves proof of CRF pathway correction (Majerová et al., 2022; Spada et al., 2022). Recent breast cancer feasibility data suggest that bilateral taVNS for insomnia can reduce fatigue scores and increase nightly HRV, but the study is preliminary and sleep-focused rather than a CRF-specific target-validation trial (Do et al., 2026). A pilot protocol has also extended taVNS testing to fatigue in gastrointestinal cancer, although it remains a feasibility-stage study rather than evidence of pathway correction (Gierthmuehlen et al., 2023). A recent breast cancer tVNS review similarly frames neuromodulation as a neuroimmune-autonomic strategy for survivorship symptom clusters, while correctly noting that CRF-specific pathway testing is still absent (Do and Tyler, 2026). Table 4 summarizes target classes, candidate phenotypes, intervention examples, pharmacodynamic biomarkers, and the falsification criteria that distinguish target engagement from clinical translation. The falsification column is included because pathway-directed studies should state in advance what result would weaken the mechanistic claim.

**Table 4:** Molecular target-validation matrix for gut-derived immune–metabolic signaling in CRF.

Target class	Best-fit biological phenotype	Intervention examples	Core pharmacodynamic readouts	Falsification criterion	Key supporting references
Barrier restoration and mucosal anti-inflammatory support	Active chemotherapy/radiotherapy, mucositis or diarrhea, elevated barrier/inflammatory markers, physical fatigue dominant.	SCFA restoration, fermentable fiber, selected probiotics/synbiotics, glutamine-like nutritional support, mucosal protectants.	Permeability/tight-junction markers, LBP/sCD14, mucosal or plasma cytokines, GI toxicity score, actigraphy/FACIT-F physical.	GI symptoms improve but fatigue and target biomarkers do not; fatigue improves without barrier or inflammatory target engagement.	Sonis (2004), Dahlgren et al. (2023), and Yonekura et al. (2022)
SCFA/FFAR/HDAC axis	Low fecal/plasma SCFAs or reduced butyrate-producing function; preserved ability to tolerate fermentation.	Prebiotics, defined consortia, butyrate/tributyrin, dietary programs designed around fermentability.	Stool metagenomic function, fecal and plasma SCFAs, histone acetylation or inflammatory tone where feasible, HRV.	Microbial composition changes without SCFA, barrier, autonomic or fatigue effect.	Guo et al. (2023), Xiong et al. (2023), Otto-Dobos et al. (2024), Dalile et al. (2019), and Goswami et al. (2018)
Tryptophan-kynurenine and indole-AHR axis	High Kyn: Trp or unfavorable kynurenine profile; inflammatory fatigue, depressive symptom overlap, barrier injury with low protective indoles.	Anti-inflammatory and exercise/nutrition strategies; carefully justified IDO/TDO/KMO-informed studies; formula/probiotic studies with targeted indole panels.	Trp, Kyn, Kyn: Trp, downstream kynurenines, targeted indoles, AHR target genes, IL-22/barrier markers.	Kynurenine or indole biomarkers change without fatigue-domain improvement, or fatigue improves in a biomarker-negative subgroup.	Molfino et al. (2024), Yan et al. (2024), Lanser et al. (2020), Holthuijsen et al. (2024), Holthuijsen et al. (2025), and Kim et al. (2023)
Enteroendocrine and 5-HT3 gut-sensory interface	Fatigue tightly coupled to nausea, appetite disturbance and gut sensory symptoms during chemotherapy.	5-HT3-related strategies, antiemetic-phenotype designs, mucosal metabolite modulation.	Separate nausea and fatigue scores, antiemetic exposure, EC/5-HT markers where feasible, vagal/NTS activation in models.	Apparent fatigue effect disappears after adjusting for nausea, appetite or sleep.	Goswami et al. (2018), Kaelberer et al. (2018), Chiang and Ferrell (2020), and Bellono et al. (2017)
Vagal and cholinergic anti-inflammatory modulation	Low HRV, inflammatory phenotype, active gut symptoms, absence of dominant anemia/cachexia.	taVNS, HRV biofeedback, exploratory α7nAChR strategies in preclinical settings.	HRV, inflammatory markers, stimulation adherence, sham-controlled symptom response, optional brainstem fMRI.	Symptom change occurs without autonomic or inflammatory engagement, or target engagement occurs without fatigue effect.	Tracey (2007), Breit et al. (2018), Bonaz et al. (2018), Do et al. (2026), Gierthmuehlen et al. (2023), and Do and Tyler (2026)
Brainstem/DRN discovery-stage targets	Preclinical models with demonstrated gut biological engagement and NTS/DRN activation linked to fatigue-like behavior.	Projection-specific chemogenetics/optogenetics, receptor pharmacology in models; no routine clinical use yet.	NTS c-Fos/photometry, DRN 5-HT dynamics, projection tracing, activity/effort behavior with sickness controls.	Manipulating the node does not modify fatigue-like behavior after peripheral biological engagement is established.	Ran et al. (2022), Holt (2022), Ren et al. (2019), and Hamada et al. (2024)
Multi-component medicinal plant/supportive formulation (target-engagement probe)	Advanced cancer CRF or myelosuppression-associated fatigue; subgrouping by hemoglobin, gut biomarkers, Kyn: Trp and HRV.	Add-on randomized trials; animal studies with antibiotics/FMT/vagotomy and pharmacodynamic readouts.	Hematopoiesis, inflammatory markers, SCFAs/indoles/Kyn: Trp, barrier markers, HRV/NTS readouts, quality-control fingerprints.	Benefit without gut, autonomic or metabolic engagement suggests hematopoietic or nonspecific supportive effects rather than gut–brain mediation.	Gu et al. (2024), Song et al. (2021), Mo et al. (2025), Zeng et al. (2024), and Dai et al. (2025)

In this table indicate representative evidence supporting each target class and do not imply that all interventions are clinically ready for CRF. Brainstem/DRN mechanisms remain at the discovery stage and require target validation and causality testing before efficacy is interpreted mechanistically. α7nAChR, alpha7 nicotinic acetylcholine receptor.

## 9 Molecular interpretation of multi-component supportive interventions

Multi-component supportive interventions raise a specific problem for molecular interpretation. They may be clinically relevant, but their effects cannot be assigned to a gut–brain pathway unless the product is batch-characterized and biological engagement is measured. Complex multi-component traditional formulations such as Fufang E’jiao Jiang (published elsewhere as Fufang E’jiao Syrup; FEJ) are therefore not used here as evidence for the proposed gut-derived mechanism. They are discussed only as examples of the level of target validation required when a complex supportive intervention is tested for CRF.

For such interventions, improvement in a total fatigue score is not sufficient for mechanistic interpretation. A prespecified molecular or physiological target should change in the expected direction. In a hematopoietic phenotype, this may involve hemoglobin and myelosuppression-related markers. In a gut-derived immune–metabolic phenotype, it would require barrier or translocation markers, SCFA or tryptophan-metabolite panels, inflammatory readouts, HRV, or brainstem engagement markers. Target engagement should precede symptom improvement. If fatigue improves without gut, autonomic, or metabolic engagement, the result should be interpreted as supportive or hematopoietic rather than gut–brain mediated. Preclinical and clinical studies of such interventions should therefore distinguish hematopoietic support from gut-derived signaling. Preclinical models should measure food intake, body weight, tumor burden, hemoglobin, activity, and effort behavior alongside gut and brain readouts. Antibiotic depletion, FMT, or germ-free recipients can test microbiome dependence; vagotomy or selective vagal silencing can test vagal dependence; receptor antagonism or genetic tools can test candidate metabolite sensors where feasible. Quality control is central because complex formulations can combine animal-derived and botanical materials, and traditional-medicine quality-control principles remain essential when such formulations are used for mechanistic testing (Xie and Leung, 2009; Zhang W. et al., 2019). Figure 4 is intended as a biomarker-guided validation scheme for complex supportive interventions, not as a mechanistic claim for any single product. Clinical signals from randomized trials of FEJ for CRF have been reported as abstracts and protocols. Broader meta-analytic work on traditional Chinese medicine approaches for CRF has also reported clinical effects, but these studies do not establish the mechanism of any specific formula. Preclinical studies of FEJ have documented hematopoietic effects (Liu et al., 2014; Zhang Y. et al., 2019). These observations support clinical and biological plausibility, but they do not prove a gut-derived mechanism. The same logic applies to any multi-component intervention proposed for CRF: a clinical signal establishes potential, preclinical data support plausibility, but only target-engagement experiments with matched biomarker readouts can establish a molecular mechanism.

**Figure 4:**
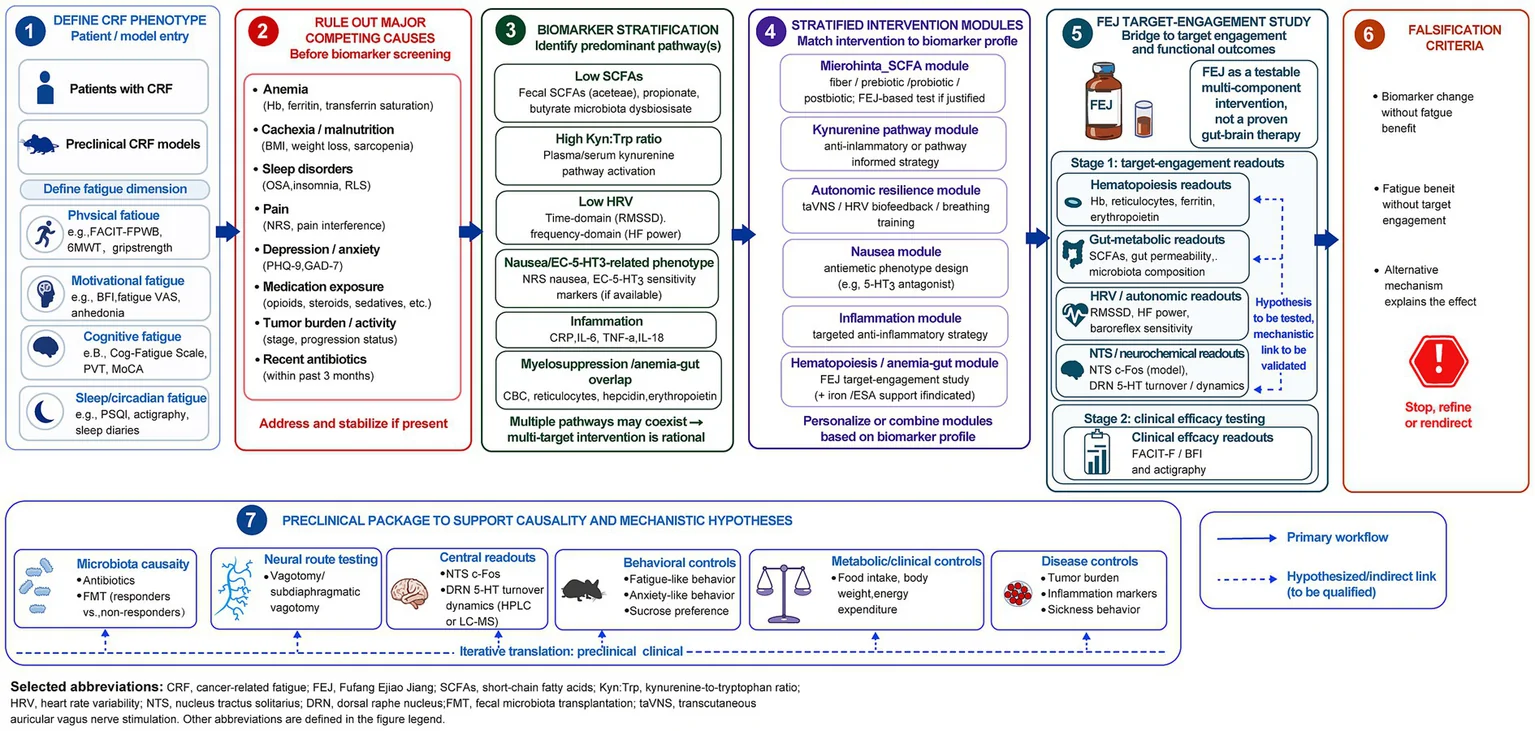
Biomarker-guided trial design and target-engagement program. The workflow begins with CRF phenotype definition and screening of major competing drivers, followed by biomarker stratification for gut injury/low SCFAs, high Kyn: Trp, low HRV, nausea/EC-5-HT3-related features, inflammatory status, and myelosuppression/anemia–gut overlap. Multi-component interventions are positioned as testable probes within a broader biomarker-guided framework. Dashed arrows indicate hypotheses and mechanistic links that require validation before efficacy claims receive mechanistic interpretation. Plant-derived interventions are positioned as probes whose proposed microbiota-dependent step must be demonstrated before a gut-mediated mechanism is claimed.

## 10 Discussion: molecular target validation and future directions

The main implication of this framework is that CRF studies should begin with a molecular hypothesis rather than a generic intervention category. A barrier-directed intervention should show reduced microbial exposure or improved epithelial integrity. A fermentation-directed intervention should show altered SCFA availability and host sensing. A tryptophan-pathway study should define whether it targets kynurenine activation, microbial indole-AHR signaling, or enterochromaffin-cell serotonin biology. A vagal intervention should show autonomic or brainstem engagement. A plant-derived intervention should demonstrate the predicted microbiota-dependent event—such as microbial community or functional remodeling, or biotransformation of the parent compound—together with the resulting mediator and host target engagement, rather than merely an improvement in a symptom score. Without this first layer of evidence, symptom improvement cannot be assigned to a gut-derived pathway.

Future CRF studies should retain conventional clinical discipline. Baseline assessment should define cancer type, treatment stage, anemia, cachexia, depression, sleep disturbance, pain, medications, antibiotic exposure, diet, and gastrointestinal toxicity. Fatigue should be measured by domain rather than total score alone. A useful clinical structure is a two-stage design. Stage 1 demonstrates pathway engagement in an enriched subgroup: a barrier intervention should lower translocation markers or improve permeability indices; a fermentation intervention should alter SCFA availability and related host readouts; a vagal intervention should change HRV or another autonomic marker. Stage 2 then tests whether clinical fatigue improves in the same biological subgroup. Failure at Stage 1 is a target-engagement failure; failure at Stage 2 is a clinical-translation failure. Separating these outcomes would make negative and positive trials more interpretable.

Adaptive designs are well suited to this problem. A run-in phase can characterize diet, microbiome function, gut symptoms, inflammation, HRV, and fatigue domain. Patients can then be assigned to the intervention most consistent with their biology. This is more demanding than a conventional parallel-group trial, but it matches the biology of CRF more closely and is more likely to yield interpretable results. Preclinical validation should also precede large clinical claims. A comprehensive preclinical assessment includes treatment-induced gut injury, longitudinal behavior, stool and plasma metabolomics, mucosal barrier assays, vagal or humoral route testing, and brainstem readouts. FMT, antibiotics, germ-free recipients, and vagotomy can test causality, but each has interpretive limits: antibiotics can alter behavior independently of microbiota, germ-free animals have abnormal immune and neural development, and vagotomy affects motility, appetite, and inflammation. These tools are strongest when combined with rescue experiments, pathway-specific biomarkers, and appropriate reporting standards for animal and fecal-transfer studies (Hooijmans et al., 2018; Secombe et al., 2021). Used in isolation, they can replace one set of confounds with another.

Biomarker stratification remains provisional. Low SCFAs, high Kyn: Trp, low HRV, barrier injury, and altered brainstem activity are candidate enrichment variables, not established companion diagnostics. Their value depends on reproducibility, temporal stability, treatment responsiveness, and association with a fatigue domain. Multi-omics can identify candidate mediators, but correlation networks are not mechanisms. A metabolite that correlates with fatigue may reflect diet, tumor burden, renal or liver function, drug exposure, muscle wasting, or infection. Without perturbation, rescue, time ordering, and route-specific readouts, an omics dataset cannot distinguish among these possibilities. The most useful role of omics in this field is to nominate smaller sets of measurable signals for intervention rather than to produce larger descriptive networks.

## 11 Limitations

Several limitations should temper the framework proposed here. First, direct causal evidence linking gut-derived molecular signals to CRF remains limited. Much of the mechanistic support comes from mucositis biology, inflammatory models, microbiome research, and basic gut–brain studies rather than from CRF-specific experiments. Direct evidence that medicinal plant-derived compounds improve CRF specifically through microbiota-dependent mechanisms is even more limited, since most plant-side support derives from pharmacokinetic, neuropsychiatric, and non-oncology models. Second, fatigue-related animal behavior does not fully reproduce the human symptom. Reduced activity, reduced effort, and sickness behavior should be separated where possible. Third, peripheral biomarkers such as fecal SCFAs, plasma Kyn: Trp, LBP, sCD14, or HRV are useful engagement markers, but none of them alone proves a gut-to-brain causal route. Fourth, multi-component interventions create attribution problems. Without batch characterization, pathway-specific biomarkers, and route-testing experiments, clinical improvement should not be interpreted as proof of a defined molecular mechanism. Statements about company-related products, including Fufang E’jiao Jiang (FEJ), follow the standard set out in Section 9 and carry no mechanistic claim.

## 12 Conclusion

CRF is unlikely to be explained by a single gut-derived pathway, and medicinal plant-derived compounds should not be exempted from this standard merely because they are natural. The more useful question is which molecular signal is present, which host sensor detects it, which route carries it, and which fatigue domain is affected. The injured intestinal mucosa provides a plausible interface where microbial metabolites, MAMPs, bile acids, and enteroendocrine signals can be converted into immune, endocrine, and neural inputs; medicinal plant-derived bioactive compounds enter this interface through three non-exclusive routes—reshaping the microbial community, undergoing microbial biotransformation, and acting directly on host targets—of which this review emphasizes the two microbiota-dependent routes. SCFAs, tryptophan-derived metabolites, bile-acid signals, and vagal or humoral pathways are therefore best viewed as candidate mediator–sensor modules rather than established CRF mechanisms. The next step is not broader symptom-only trials, but biomarker-matched studies that test target engagement, including the microbial conversion of plant-derived substrates, before mechanistic interpretation. This approach can help distinguish gut-derived fatigue biology from anemia, cachexia, sleep disturbance, pain, mood symptoms, and nonspecific supportive effects.

## Glossary

5-HT: 5-hydroxytryptamine
5-HT3: 5-hydroxytryptamine receptor 3
AHR: aryl hydrocarbon receptor
BFI: Brief Fatigue Inventory
CAZyme: carbohydrate-active enzyme
CCK: cholecystokinin
CNS: central nervous system
CRF: cancer-related fatigue
CRP: C-reactive protein
DRN: dorsal raphe nucleus
DVC: dorsal vagal complex
EC: enterochromaffin cell
ERβ: estrogen receptor beta
FACIT-F: Functional Assessment of Chronic Illness Therapy–Fatigue
FACT-Cog: Functional Assessment of Cancer Therapy–Cognitive Function
FEJ: Fufang E’jiao Jiang
FFAR2/FFAR3: free fatty acid receptor 2/3
FMT: fecal microbiota transplantation
FXR: farnesoid X receptor
GLP-1: glucagon-like peptide 1
HCAR2: hydroxycarboxylic acid receptor 2
HDAC: histone deacetylase
HK: 3-hydroxykynurenine
HPA: hypothalamic–pituitary–adrenal
HRQoL: health-related quality of life
HRV: heart rate variability
IDO1/IDO2: indoleamine 2,3-dioxygenase 1/2
IL-6: interleukin-6
IL-22: interleukin-22
KA: kynurenic acid
KAT: kynurenine aminotransferase
KMO: kynurenine 3-monooxygenase
Kyn: kynurenine
LBP: lipopolysaccharide-binding protein
LC: locus coeruleus
LPS: lipopolysaccharide
MAMPs: microbe-associated molecular patterns
ME/CFS: myalgic encephalomyelitis/chronic fatigue syndrome
NTS: nucleus tractus solitarius
PBN: parabrachial nucleus
PROMIS: Patient-Reported Outcomes Measurement Information System
PSQI: Pittsburgh Sleep Quality Index
PYY: peptide YY
QA: quinolinic acid
sCD14: soluble CD14
SCFAs: short-chain fatty acids
SII: systemic immunity-inflammation index
SNRI: serotonin–norepinephrine reuptake inhibitor
SSRI: selective serotonin reuptake inhibitor
TDO: tryptophan 2,3-dioxygenase
TGR5: Takeda G protein-coupled receptor 5
TLR4: Toll-like receptor 4
Trp: tryptophan
taVNS: transcutaneous auricular vagus nerve stimulation
VTA: ventral tegmental area
α7nAChR: alpha7 nicotinic acetylcholine receptor.
